# Can Psychiatry be Misused Again?

**DOI:** 10.3389/fpsyt.2013.00101

**Published:** 2013-09-09

**Authors:** Adonis Sfera

**Affiliations:** ^1^Psychiatry, Patton State Hospital, South Coast Clinical Trials, Anaheim, California, CA, USA

**Keywords:** psychiatry, genetics, eugenics, legal case precedent, genetic testing

## Introduction

The Human Genome Project was completed in 2003. While the medical community is still digesting the results, some questions are already emerging:

1. Are psychiatrists prepared to discuss with patients and families the genetic aspect of mental illness (1)?
2. Since genetic testing reflects probabilities and risk factors for mental illness, are patients and families able to understand that genes do not cause diseases or symptoms, but rather “conspire” with the environment to bias the individual toward a syndrome or symptom?
3. Is the data relevant enough to be communicated even in the absence of adequate treatments?
4. Do the offspring of mentally ill patients have the right to know the results of their genetic testing? What about the right not to know if they choose so?
5. Can insurers or employers misuse genetic information?
6. Are we headed toward a twenty-first century neo-eugenics?

## Historic View of Psychiatry

Historically, psychiatry was frequently misused for political or economic gain. Science of eugenics emerged at the beginning of last century (2). It stated that the genetic pool of the population could be improved by limiting the reproductive rights and even survival of the “unfit” individuals. Psychiatrists played a crucial role in the eugenic movement in Nazi Germany. In September 1939, Hitler signed a document called “The Law for the Prevention of Offspring with Hereditary Diseases.” This law specified three actions to prevent the perpetuation of hereditary diseases in the German population: mental retardation, schizophrenia, and alcoholism. These actions were:

1. Forced sterilization of “unfit” population.
2. Children with deformities and other hereditary disorders were reported to a central registry from where a committee of academics decided who would be killed.
3. “Action T4” was a program that targeted adult psychiatric patients for extermination (3).

This euthanasia program entailed killing of patients by gas in special hospitals in the years 1939–1941, and in psychiatric hospitals in the years 1942–1945. In this latter period, patients were killed with lethal injections and through the introduction of a starvation diet.

It is interesting that the elite of German psychiatry such as university professors and hospital directors decided whether a given patient would meet the criteria for the euthanasia program (3, 4). These criteria were met by individuals who:

1. Had specific mental disorders and were unable to work, or
2. Could carry out purely mechanical tasks, or
3. Had continuously spent at least 5 years in an asylum, or
4. Were kept under custody as criminally insane, or
5. did not possess German citizenship, or
6. Were not of the German or similar races (5, 6).

### American eugenics

Prior to World War I eugenics was widely accepted in the U.S. academic community. The American eugenics movement was rooted in the biological determinist ideas of Sir Francis Galton. Galton coined the term “eugenics” in 1883. The essence of the concept was described in his book “Hereditary Genius,” published in 1869. Galton believed that through selective breeding human species should direct its own evolution. Others who followed Galton in principle pursued a difference approach, advocating involuntary sterilization and restrictive laws for marriage and immigration, a program often labeled “negative eugenics.” Focusing first on the mentally ill and mentally handicapped, negative eugenics expanded to embrace notions of racial inferiority (7).

In 1927, the U.S. Supreme Court had empowered the states to determine who should and should not be permitted to reproduce. From this point on eugenics was enforced by state laws. These family laws prohibited the marriage of “lunatics,” “imbeciles,” “epileptics,” the “insane,” and the “weak minded.” Some of these laws lingered in different states of the union in one way or another until 1980s (8, 9).

Between 1907 and 1940 a total of 18,552 insane individuals were sterilized in the United States. Half of the procedures were done in California where the superintendent of the Stockton State hospital believed that marriage licenses in the general population should not be given to anyone with “a taint of insanity in his or her family” unless the person had first undergone sterilization (10).

Psychiatry's desire for greater respectability in the medical profession made eugenic “science” attractive. Nathan Hale wrote in “Freud and the Americans”: “Logically, only eugenics programs could halt the apparently mounting incidence of insanity.” Barbara Sicherman, in “The Quest for Mental Health in America: 1880–1917” similarly observed that “most psychiatrists were greatly interested in the scientific study of eugenics” (10).

## Psychiatry in Recent History

Psychiatry was also misused in more recent history. For instance, in 1969 Soviet academic psychiatrists led by Snezhnevsky and his colleagues coined the term sluggish schizophrenia (11, 12). This notion allowed for the diagnosis of schizophrenia to be made even in the total absence of signs and symptoms of psychosis. Because of this reason dissidents and political prisoners were confined in psychiatric hospitals where they were mistreated by being ordered unnecessary ECT without anesthesia, large doses of antipsychotic medications and insulin comas. In response to these widely publicized reports of the misuse of psychiatry in the Soviet Union for the repression of political dissent, the Soviet All-Union Society of Neurologists and Psychiatrists was excluded from the World Psychiatric Association in 1984 (11, 12).

Even more recently, Radovan Karadzic, an academic psychiatrist and leader of Bosnian Serbs in former Yugoslavia ordered “ethnic cleansing” (extermination) of Bosnian Muslims and Croats in the early 1990s. Mr. Karadzic is currently accused of crimes against humanity and is awaiting trial by the international tribunal in Hague (13, 14).

## Conclusion

The cultural legacy of eugenics might influence the development of psychiatric genetics. Is psychiatry in danger of being misused again? A commonly expressed fear is that genetic information could be used for harm rather than for good. There is considerable concern about the privacy of genetic information and its potential use for discriminatory purposes by insurance companies and employers. Will genetic information, result in a portion of the society that will have sufficient risk factors as to be refused employment, medical, or life insurance because the insurance premiums would be too high for the employers to support? Will pharmacogenetics by the same token disclose strata of population that does not respond to available medication rendering their treatment “too expensive” for the insurers or the government? For instance individuals with the short allele for serotonin transporter are at higher risk of depression in a stressful environment and therefore at risk of missing work more often than individuals with long allele. Would they be less likely to obtain employment or medical insurance if the employer or the government would have access to their genetic data? Would this population be marginalized and perhaps persecuted based on their genetic information?

The grass root psychiatrists are not well informed of the developments in genetics. This puts us at risk of being misused. In the above example, for instance, would a psychiatrist have the duty to warn family members that they might be at risk of depression? What about a patient with Alzheimer's disease who is positive for the ApoE gene? Do we warn the children of the patient of their potential risks for Alzheimer's or do we consider the family members as not being under our care and thus do not have the obligation to disclose this risk? The same could apply to somebody with Velocardiofacial syndrome at high risk for schizophrenia.

In the last decade several courts throughout the US have allowed claims to proceed against a physician based upon an alleged failure by the physician to notify family members that a patient has an inheritable disease (15). In a legal precedent (16) in 2004 the mother of a daughter with fragile X syndrome claimed that her child's physician failed to inform her that she might be at risk of conceiving children with the same condition. The Minnesota Supreme Court adjudicated the case in favor of the mother.
